# “METAPAD” (METAbolic PAthways Decoded) – a gaming innovation to ease the complexity of metabolic pathways by promoting self-directed, active, participatory learning in small groups

**DOI:** 10.1186/s12909-023-04587-5

**Published:** 2023-08-25

**Authors:** Krishna Mohan Surapaneni

**Affiliations:** 1 Department of Biochemistry, Panimalar Medical College Hospital & Research Institute, Varadharajapuram, Chennai, 600-123 Tamil Nadu India; 2 Department of Medical Education, Panimalar Medical College Hospital & Research Institute, Varadharajapuram, Chennai, 600-123 Tamil Nadu India

**Keywords:** Puzzle-based approach, Self-directed learning, Biochemistry, Metabolic pathways, Active student engagement

## Abstract

**Background:**

In medical education, the conventional didactic lectures make the learning process tedious and cause disinterest among students. To overcome this, an effective experiential learning integrated with game-based approach that intends to make the learning process fun filled, interesting and enhance the active participation of students to understand complex topics in a simple and optimum manner should be adopted.

**Objectives:**

The purpose of this study is to assess the efficiency of a puzzle-based innovation, METAPAD (METAbolic PAthways Decoded) in understanding the complexity of biochemical pathways by enhancing the self-directed learning of students through active engagement in small groups

**Methods:**

In this mixed-method study, 103 first professional year medical students in small groups were enrolled for the METAPAD gaming puzzle-based learning activity. The puzzles were integrated with a relevant clinical case study. Decoding the puzzle after identification of the metabolic pathway involved in the case was conducted in level 1,2 and 3 with increasing complexity of puzzles at every level. Following the puzzle activity, A 21-item questionnaire was administered to evaluate the usefulness of the innovation and students’ perceptions towards different learning styles. Also, students’ feedback was obtained through personal interviews for qualitative analysis and thematic analysis was performed.

**Results:**

One hundred and three first year undergraduate medical students participated in the study. Most of the students perceived the METAPAD gaming puzzle to be an effective and innovative style of learning. There was not any significant association between age, gender and acceptance towards the METAPAD gaming puzzle. The predominant type of learning style among the students was multimodal (49.5%). Also, there was no great influence of the learning styles on the overall perception towards the METAPAD gaming puzzle. However, learners emphasized the need to focus more while solving the puzzles.

**Conclusion:**

Students perceive the METAPAD gaming puzzle as an actively engaging teaching-learning method that enhances their creativity, critical thinking and problem-solving abilities. It promotes self-directed learning of complex pathways in biochemistry which helps in remembering and recalling information and improves their learning skills.

**Supplementary Information:**

The online version contains supplementary material available at 10.1186/s12909-023-04587-5.

## Introduction

The course of medical education is designed in such a way that students need to learn ‘a lot’ in ‘very short’ periods of time. Conventional and monotonous methods of teaching certainly do not motivate the students to learn but cause mental stress and burden of syllabus adversely affecting their academic performance. In order to inculcate right knowledge in precise and effective manner, the National Medical Council (NMC) has reformed the traditional Medical Education into Competency-Based Medical Education (CBME) curriculum that recognises the important areas of focus and categorises them under ‘competencies with specific learning objectives’ [1]. This enhances the learning process of students by paying attention to clinically oriented topics that improves the interpretation and problem solving abilities. But teaching these competencies in an effective and innovative way to foster active student engagement and best learning outcomes is a challenge to all medical educators. The Panimalar Medical College Hospital & Research Institute, established in 2021 strives to deliver the best educational service by introducing various curriculum innovations to foster active student engagement and promote self-directed learning experiences.

As the students enter their medical profession, it is vital to acclimatise them to the busy environment and inculcate curiosity towards the subjects by motivating their learning to achieve optimum benefits of teaching. The first year of medical curriculum comprises of Anatomy, Physiology and Biochemistry as core subjects. All the three are fundamental medical subjects with vast concepts that are highly crucial for medical students to master. The framework of the curriculum is in such a manner that, anatomy sessions have well planned practical demonstrations with human cadavers that make learning new terms and remembering anatomical locations easier as students get the opportunity to explore every part of human body through dissection. Although physiology lectures are more of macroscopic concepts, the microscopic functioning forms the foundation of human physiology and a clear understanding that biochemical alternations will lead to physiological changes is essential. Thus, the challenge lies in imparting the concepts of biochemistry in the most effective way as it forms the basis of life, explaining the molecular mechanisms of all physiological and pathological conditions.

Life of all forms depends on biochemical reactions and processes. Biochemistry is the most influential fields of science having indispensable contribution to research and medicine which integrates the biological and chemical functions of cells upon which all metabolic cycles are built [2]. In order to understand the complexity of life, we must understand the complexity of metabolic pathways and hence it is mandatory that every medical student has a strong foundation in biochemistry. But the intricate pathways with extensive enzymes and compounds vocabulary makes it difficult for the students to comprehend and recall during examination in a conventional classroom teaching environment as they cannot visualise the complex molecular reactions. Thus to facilitate students’ learning, an innovative puzzle-based approach was introduced to understand the metabolic pathways in biochemistry that facilitates experiential learning of the molecular basis of life effectively.

## A game-based innovation

The students of medical education for a long term have been describing their learning to be ‘hectic and enormous’ and thus call for a more student-friendly academic curriculum [3]. To alleviate their stress and make learning more enjoyable, educators are focusing an experiential learning strategy that focuses on creating fun-filled and innovative interventions to encourage the students to engage in active discussion and self-directed learning methods. Innovations in Medical Education could be defined as, “the implementation of selected new methods in education that will cause an overall improvement’. Gamification is one such innovation that aims to integrate knowledge with fun. It creates an environment where students learn with creativity and enthusiasm. However, the effectiveness of all games in imparting right knowledge is uncertain [4]. The literature search has identified that game-based innovations provided promising results in undergraduate medical students in terms of motivating the students to learn effectively by participating in small groups which enhances their interaction and teamwork skills [5, 6]. Among the different games, solving puzzles was identified as an effective and interesting method of learning and understanding concepts [7].

A puzzle is a fun, novel, not too easy and not too hard but tricky form of play [8]. Learning through puzzles has proven to improve the memory, planning and problem solving skills of students [9]. It provides a wholesome exercise to the brain that combines visuo-spatial ability to capture small and intricate details of any problem. Puzzles are also ‘Stress Busters’ that relaxes the mind by causing happiness which improves productivity at work or school and promote better collaboration and teamwork among the users [10].

Unfortunately, much of focus has not been given in adoption of this innovation for learning biochemistry among undergraduate medical students. Thus, recognising the impact of game-based learning and that metabolic pathways play a crucial and critical step in understanding the molecular, cellular evolution and the existence of life, a unique fun program titled “METAPAD” (**META**bolic **PA**thways **D**ecoded) was developed which aims at making the undergraduate students of healthcare professions to understand the complex metabolic pathways and their clinical significance with ease. This is an innovative activity based teaching-learning methodology that promotes self-directed, active learning in small groups, guiding and engaging students in achieving the agreed learning outcomes with the introduction of the component of game-based learning.

### Research question

This study aims to address the following research questions :

1. How does METAPAD puzzle-based activity promote active learning of complex metabolic pathways in biochemistry for Phase 1 MBBS students?
2. Do individual learning style preferences have an influence over how students perceive METAPAD to be an effective learning tool?

### Objectives

The intended objectives of this study are (1) To design a fun-filled puzzle program that strengthens the concepts of biochemistry by learning of metabolic pathways (2) To enhance the critical thinking and problem-solving skills of students through clinical case interpretation, (3) To facilitate self-directed learning through active student engagement in small groups, (4) To understand the effectiveness of activity-based learning compared to didactic lectures and (5) To know the students’ perceptions of METAPAD gaming puzzle as a useful study tool and analyse the perceptions in association with different learning styles.

### Designing of the “metapad” gaming puzzle – the conceptual framework

Based on the critical review of the literature and analysing the existing problems in teaching-learning methods, Our innovation, METAPAD ( METAbolic Pathways decoded) gaming puzzle has been built upon the two theories for the educators and learners for effective transfer of knowledge and optimum learning by students are :

1. **Constructivist Theory** by *Jerome Bruner* [11] – [To enhance teaching]
2. **Experiential Learning Theory** by *David Kolbe* [12] – [To enhance learning]

The first theoretical framework explains the role of instructors in effectively transferring the knowledge. The second theory explains how ‘learning by doing’ enhances the learning and retaining of information. In the first component, instructors or educators are the main point of focus. Brunner’S theory deals with four main areas to deliver knowledge, 1) Inculcating the tendency to learn, 2) designing the body of knowledge for structured delivery so that students can easily grasp 3) efficient method of presenting the content and 4) Rewarding and punishing [11]. The second component explains that students or learners placed at the core of circle and explains the process of learning by doing. It states that learning is a process whereby knowledge is generated by conversion of experience. It is composed of four aspects 1) Concrete experience, 2) reflective observation, 3) abstract conceptualisation and 4) active experimentation [12]. Thus ‘The Constructivist Theory’ was adopted as the first component to develop effective teaching methods to address the issues in delivering complex concepts in biochemistry and ‘The Experiential Learning Theory’ as the base to enhance the learning in a fun filled and engaging activities like games and puzzles that I love the students to learn and understand complex metabolic pathways in an interesting and experiential manner.

Keeping in view the existing issues with conventional classroom teaching and the need to implement experiential fun filled learning strategies, the **‘METAPAD- META**bolic **PA**thways **D**ecoded**”** Gaming Puzzle was proposed to understand the complexity of metabolic pathways in biochemistry and make learning and easy and joyful experience. METAPAD gaming puzzle combines the theories of teaching and learning to effectively deliver knowledge and optimum grasping of that information through experiential learning. Thus this game-based approach clearly satisfies the competencies of effective teaching and learning through experience and reflective ability of students.

The METAPAD gaming puzzle is designed in such a way that critical thinking, problem-solving skill, active participation, and self-directed learning are interconnected. By engaging with peers and solving clinical cases and complex puzzles, learners develop critical thinking abilities and effective problem-solving strategies. The competition and gaming environment promotes active participation and reinforces these skills and encourages a deeper understanding of the content. Moreover, learners are given the opportunity to learn on their own and also identify the contents in metabolic pathways that they would have missed learning by decoding the puzzles. This emphasis on self-directed exploration fosters autonomy, further enhancing learners’ critical thinking and self-guided problem-solving capabilities.

The components of teaching and learning achieved in METAPAD Gaming Puzzle is summarised in Tables 1 and 2.

**Table 1:** Components of teaching covered in METAPAD gaming puzzle

Teaching component	Explanation
**Content knowledge**	The METAPAD Gaming Puzzles add developed by expert faculty with profound knowledge about the subject and effective clinical case association.
**Organisation & quality of material**	As the puzzles are built upon strong conceptual evidence it reflects the well-structured and excellence in quality of the material to provide precise and quality information to the learner.
**Teaching environment**	As METAPAD is game based teaching it inculcates interest and promptness to participate among the students so that the instructors goal to instil readiness to grasp information is achieved in students’ minds.

**Table 2:** Components of learning achieved in METAPAD gaming puzzle

Learning component	Explanation
**Experiencing**	METAPAD Gaming Puzzle promotes the experience of self-directed learning of students where they take individual responsibility of planning implementing assessing their learning experience with their prior knowledge.
**Reflecting**	Solving the METAPAD gaming puzzles help the students to reflect on the information that was taken in lectures or reading materials and improve their memory which is critical in learning the complex metabolic pathways.
**Thinking**	Solving METAPAD puzzles requires focus and clear attention to detail students are able to pay attention to intricate details of the puzzle that enhances their ability to think critically and solve problems.
**Acting**	As METAPAD gaming puzzle is conducted as a group activity students are motivated and actively engaged in small groups to act promptly to solve the puzzle by collaborating with the team members which enhances their interaction and skills of working together.
**Interpersonal skills development**	Active participation in METAPAD gaming puzzles not only inculcates subjective knowledge but improves the critical and lateral thinking ability of students enhancing their cognitive flexibility and creativity in providing solutions through problems.

## Materials and methods

### Study design and study participants 

This is a mixed-method experimental study conducted at Panimalar Medical College Hospital & Research Institute, Varadharajapuram, Poonamallee, Chennai – 600 123, Tamil Nadu, INDIA. This approach was followed as it allows for a comprehensive assessment of the innovation’s impact by combining the analysis of participants’ experiences and perceptions both quantitatively and qualitatively. 103 First Professional Year Undergraduate medical students of the Panimalar Medical College Hospital & Research Institute, Chennai admitted in the academic year 2021–2022 were enrolled for this program (*n* = 103). Under the supervision of the faculties of Department of Biochemistry, they were randomly divided into 15 teams/groups. All the students were willing to participate in the activity. The study was conducted on November 14th, 2022. Institutional Human Ethics Committee of Panimalar Medical College Hospital & Research Institute (PMCHRI-IHEC) approval has been obtained prior to the start of the study (Approval Number: PMCH&RI/IHEC/2020/069; dated: 15.03.2022). “Written informed consent has been obtained from all the study subjects who participated in this study”.

### Organization of puzzles

**Fig. 1:**
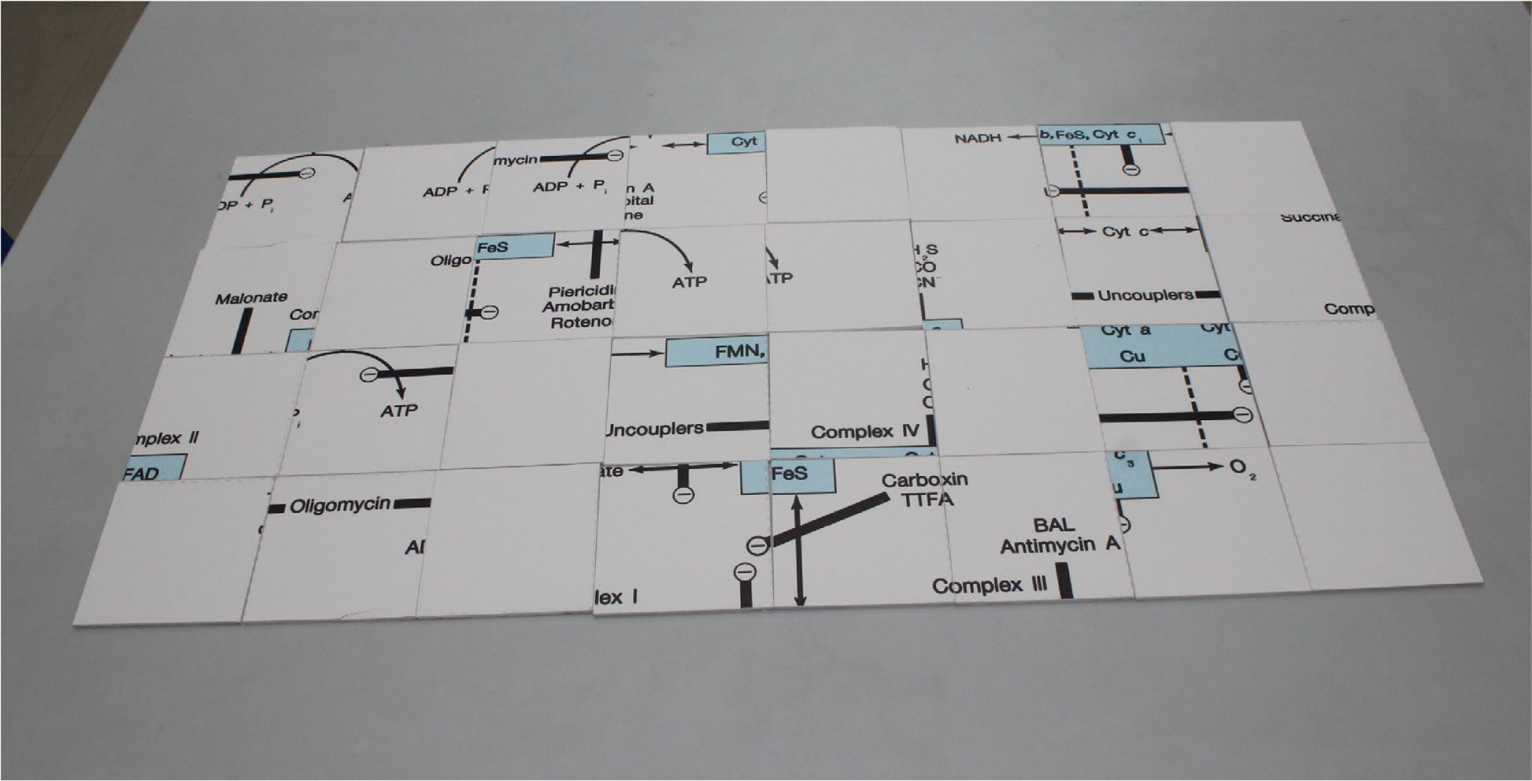
METAPAD Puzzle 1 – electron transport chain

**Fig. 2:**
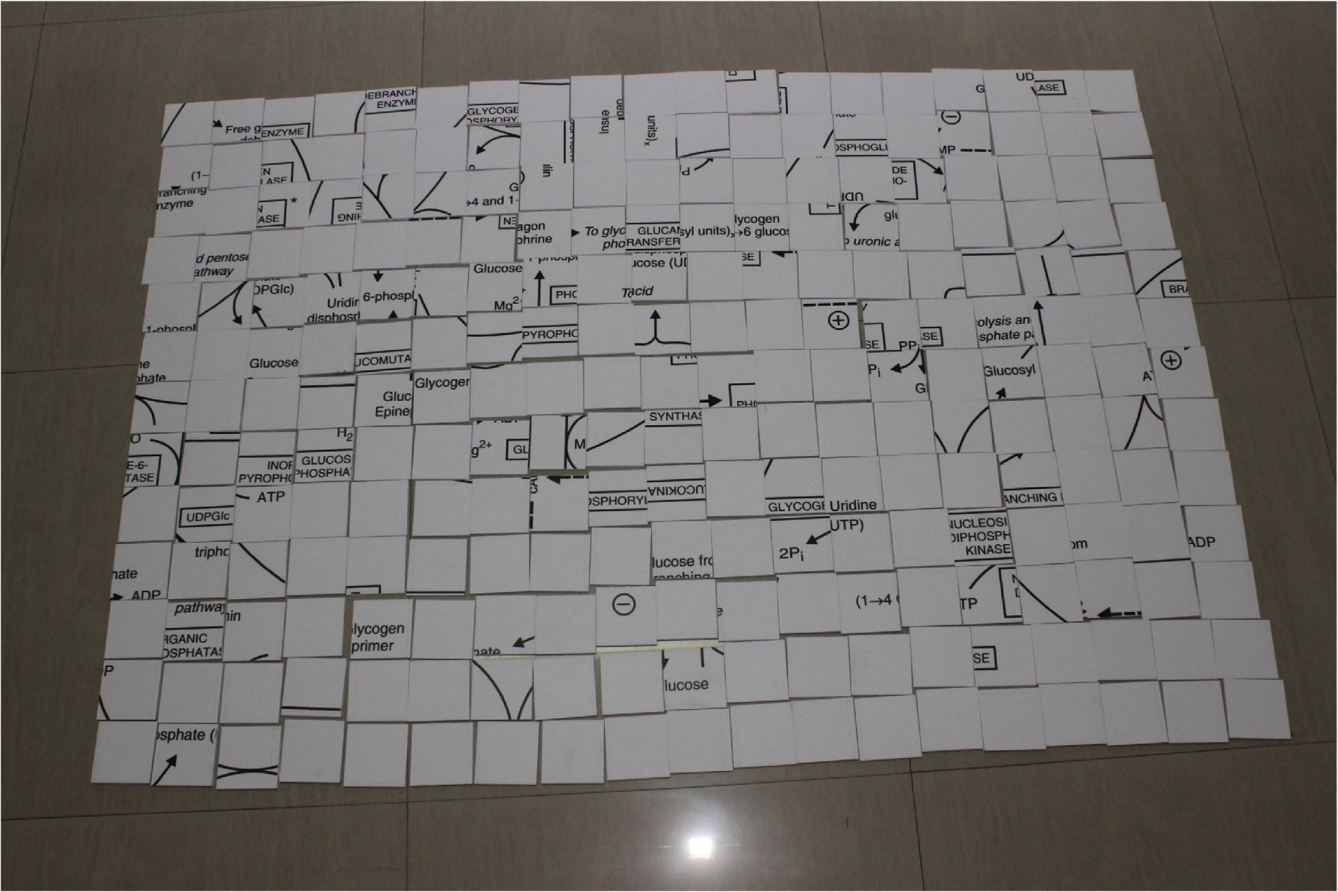
METAPAD Puzzle 2 – glycogen metabolism

**“METAPAD”** - METAbolic PAthways Decoded is a series of biochemical puzzles that comprise key metabolic pathways in biochemistry. This puzzle-based activity is divided into 3 levels: Easy, Moderate and Hard with the complexity level increasing in terms of the number of puzzle blocks in each level. Each puzzle was accompanied by a case scenario that has to be interpreted by the students and identify the metabolic pathway involved. The key pathways chosen were the electron transport chain, glycogen metabolism – including glycogenesis and glycogenolysis and cholesterol biosynthesis pathway. Table 3 depicts the level of difficulty and organisation of puzzles. Images of the METAPAD gaming puzzle are represented in Figs. 1 and 2.

**Table 3:** Organisation of puzzles

ROUND	CASE SCENARIO	METABOLIC PATHWAY INVOLVED	LEVEL OF DIFFICULTY	PUZZLE BLOCK SIZE (in inches)
1	A Person, who went into the sewage canal for clearing the obstruction, was found dead. His death was certified to be due to hydrogen sulphide gas poisoning. Decode the Metabolic Pathway involved in this case.	Electron Transport Chain	Easy	3 × 3
2	A 2-year-old boy was brought into the emergency room, suffering from severe fasting hypoglycemia. O/E and on detailed investigation, the physicians have confirmed the diagnosis of Type Ia glycogen storage disease (glucose 6- phosphatase deficiency). Simultaneously, a 3-year-old girl was brought into the emergency room, suffering from mild fasting hypoglycemia. O/E and on detailed investigation, the physicians have confirmed the diagnosis of Type VI glycogen storage disease (liver phosphorylase deficiency). Decode the Metabolic Pathways involved in this case.	Glycogen metabolism – including glycogenesis and glycogenolysis	Moderate	2 × 2
3	A 50 year old man, an executive in a corporate firm comes with the history of pain in the chest radiating to the left shoulder after a brisk walk for 10 min however the pain subsides after taking rest. (Was suspected to be suffering from Angina Pectoris). Provisional Diagnosis of Myocardial Infarction was made. (Insufficient blood supply due to narrowing of coronary blood vessels). Decode the Metabolic Pathway involved in this case	Cholesterol Biosynthesis Pathway	Hard	1 × 1

### Implementation of METAPAD

METAPAD’ 2022 was conducted in a systematic manner under the supervision of all faculty members. The puzzles were distributed to the teams. At each level, the puzzles are accompanied by a clinical case scenario. The team members will have to analyse the case, diagnose the clinical conditions and identify the metabolic pathway involved. The students’ then work together to solve the METAPAD gaming puzzle. The top 10 teams which accomplish the task by rightly arranging the puzzle in stipulated time enters the next level. The same strategy is followed for level 2 with increase in number of blocks. The top 5 teams clearing Round 2 will qualify to participate in Round 3 or the Final level. With increase in complexity of puzzles, the teams follow similar method of case study interpretation with identification of metabolic pathway and start decoding the puzzle. The winners of the activity in the 1st, 2nd and 3rd place are rewarded with prices at the end of the event to acknowledge and appreciate their active participation and teamwork.

### Assessing the perceptions of students

The students (study participants) were asked to complete two surveys. The first survey is to assess the preferred learning styles of students using The VARK – a guide to learning preferences [13]. The VARK scale categories the learning preferences into 4 groups: Visual, Aural, Read / Write, Kinaesthetic and Multimodal. The second survey was administered to evaluate the perceptions of METAPAD gaming puzzle as an effective study tool. The study participants were asked to complete a questionnaire consisting of 21-items on a 5-point Likert scale (Strongly Agree/Agree/Neither agree nor disagree/Disagree/Strongly disagree), adopted from *Ghada Bawazeer et al.* [14] and modified to assess their perception on the usefulness of the gaming based learning approach along with the integration of clinical case scenarios for understanding the complexity of metabolic pathways in biochemistry. The reliability of the tool was analysed by using Spearman’s brown prophecy formula (r) = 0.993. The items in the questionnaire are aimed at evaluating the following issues: fun in learning, problem solving skills, deep understanding and ability to recall the information, quality of puzzle material, interaction and teamwork, usability in evaluation and revision of concepts, interpersonal skills development and promotion of meaningful learning. Both the surveys were anonymous. Students were asked to complete the VARK survey first to identify their preferred learning styles and to specify it in the perception survey. Consent was obtained from all the student. Participation in the METAPAD gaming Puzzle and survey was voluntary. Students were also informed that completing the METAPAD gaming Puzzle or the survey would not avail any benefits in terms of academics or grades and is purely an innovative study to evaluate the usefulness of game based approach in enhancing the process of learning the complex metabolic pathways in biochemistry. Also for the qualitative part, students’ feedback was obtained through personal interviews and thematic analysis was performed.

Learning strategies and learning perception are chosen as factors representing the enhancement of self-directed and game-based learning because assessing how learners approach problem-solving scenarios and knowledge acquisition provides insights into the game’s effectiveness in promoting critical thinking and problem-solving skills. Additionally, understanding learners’ perceptions is crucial to evaluate their engagement and motivation, ensuring an enjoyable, valuable and meaningful learning experience that fosters active participation and self-directed learning in an evidence-informed manner.

### Statistical analysis

Descriptive analysis was conducted to report the mean and percentages and frequency for the continuous and categorical variables respectively. All the categorical variables are presented as numbers and percentages. Descriptive analysis was performed using univariate statistics to report the Mean and Standard Deviation (SD) for the continuous variable. The T-test, and Analysis of Variance (ANOVA) was performed to compare differences in the continuous variables. Mann–Whitney U test and Krushkall Wallis test were used to identify the differences in non - normal distribution. Data was analyzed and all statistical analyses were performed using Statistical Package for Social Science (SPSS, version 17) for Microsoft Windows, SPSS Inc. USA. *p* < 0.05 was considered as significant. Content analysis was performed to analyze the qualitative data collected by virtue of the students’ responses to open ended question and one to one interviews of the study participants. In addition to this, factor analysis was performed as a validity investigation to identify how accurately the perception items could measure the target variables. For this, the entire questionnaire was categorised into four domains: i) METAPAD – Game Material, ii Brainstorming with METAPAD, iii) METAPAD - Learning Experiences, and iv) Advantages of METAPAD. Principal Component Analysis for the extraction and Varimax with Kaiser Normalization for rotation ( 3 iterations) was used to perform the factor analysis. The questionnaire component with an Eigenvalue of more than 1 was deemed to be the most influencing in the particular domain under consideration for analysis. Component number and Eigenvalue were the X- and Y-axes of a scree plot graph. A rotational component matrix table was used for domains that had more than 1 component being the most influential and a component matrix table was used for domains that had only 1 component (without rotation) as the most influential to report the factor analysis results.

## Results

### Students’ perceptions towards METAPAD gaming puzzle

The data obtained from 103 first professional-year undergraduate medical students admitted at the Panimalar Medical College Hospital & Research Institute, Chennai during the Academic Year 2021–2022, who participated in the METAPAD gaming Puzzle was analysed. The mean age of the students was 19.46. Majority of the participants were females (66%). According to the VARK scale of learning styles, The predominant learning style of most of the students was multimodal (49.5%) followed by kinaesthetic (29.1%) and with the least preferred style being read/write (3.9%). Table 4 summarises the demographic characteristics of students who participated in METAPAD gaming Puzzle.

**Table 4:** Demographic characteristics of students who participated in METAPAD’2022

Characteristics	N (%)
Age ( Years)	
Minimum	18
Maximum	23
Mean	19.46
**Gender**	
Male	35 (34.0)
Female	68 (66.0)
Trans Male	0 (0.0)
Trans Female	0 (0.0)
Gender Variant/Non- Conforming	0 (0.0)
Not listed	0 (0.0)
**Learning Style**	
Visual	11 (10.7)
Aural	7 (6.8)
Read/Write	4 (3.9)
Kinaesthetic	30 (29.1)
Multimodal	51 (49.5)

The perception survey on METAPAD gaming Puzzles as an effective study tool received overwhelmingly positive responses from the students. The majority of the students enjoyed solving the puzzles and found the clinical cases interesting. They perceived the puzzles to have stimulated their curiosity in learning complex metabolic pathways in biochemistry (96.2%) and enhanced their problem-solving and critical thinking skills (> 98%). The students felt that solving METAPAD gaming puzzles helped them recall the topics they had learned (89.4%) and improved interaction among classmates (100%). They also appreciated the orientation of the puzzles towards important topics (77.6%) and believed sufficient time was given to solve them (86.4%).

Overall, the students perceived METAPAD gaming Puzzle as a valuable tool for learning and revising complex metabolic pathways (92.3%). They found it to be a good review of lecture material and believed it promoted meaningful learning compared to traditional classroom teaching (97%). The puzzles were seen to encourage active independent learning, student reasoning, and communication skills. But around 30% of the students were not sure about using the METAPAD gaming puzzle for evaluating a student. A detailed statistical analysis of the responses with regard to student’s perceptions towards the METAPAD gaming puzzle on the 5-point Likert scale with mean and standard deviation values is presented in Table 5.

**Table 5:** Students’ perceptions of METAPAD gaming puzzle as effective study tool

Perception Survey Items	Mean (SD)	Strongly Agree N (%)	Agree N (%)	Neither Agree nor Disagree N (%)	Disagree N (%)	Strongly Disagree N (%)
I have enjoyed solving the METAPAD gaming Puzzles.	4.64 (0.482)	66 (64.1)	37 (35.9)	0 (0.0)	0 (0.0)	0 (0.0)
The clinical cases given in METAPAD gaming Puzzle were interesting	4.56 (0.518)	59 (7.3)	43 (41.7)	1 (1.0)	0 (0.0)	0 (0.0)
METAPAD gaming Puzzle tool stimulates my curiosity in learning the complex metabolic pathways in biochemistry.	4.56 (0.605)	63 (61.2)	36 (35.0)	3 (2.9)	1 (1.0)	0 (0.0)
The METAPAD gaming Puzzle tool polishes my creativity.	4.44 (0.621)	52 (50.5)	44 (42.7)	7 (6.8)	0 (0.0)	0 (0.0)
The METAPAD gaming Puzzle tool enhances my problem-solving ability.	4.54 (0.556)	58 (56.3)	44 (42.7)	1 (1.0)	0 (0.0)	0 (0.0)
The METAPAD gaming Puzzles helped in improving my critical thinking ability	4.50 (0.540)	54 (52.4)	47 (45.6)	2 (1.9)	0 (0.0)	0 (0.0)
We need to be focused to solve the METAPAD gaming Puzzle	4.61 (0.528)	65 (63.1)	36 (35.0)	2 (1.9)	0 (0.0)	0 (0.0)
I am able to recall the topics while solving the METAPAD gaming Puzzle.	4.19 (0.643)	32 (31.1)	60 (58.3)	10 (9.7)	1 (1.0)	0 (0.0)
The METAPAD gaming Puzzles can be used to evaluate a student too.	3.82 (0.905)	26 (25.2)	39 (37.9)	32 (31.1)	5 (4.9)	1 (1.0)
METAPAD gaming Puzzle can increase interaction among the students.	4.70 (0.461)	72 (69.9)	31 (30.1)	0.0	0 (0.0)	0 (0.0)
The METAPAD gaming Puzzle enhanced my learning.	4.45 (0.573)	49 (47.6)	52 (50.5)	1 (1.0)	1 (1.0)	0 (0.0)
I have enjoyed classmate interaction and reviewing the content of metabolic pathways while solving the METAPAD gaming Puzzle.	4.60 (0.530)	64 (62.1)	37 (35.9)	2 (1.9)	0 (0.0)	0 (0.0)
METAPAD gaming Puzzle oriented us to the topics that we should focus on.	4.43 (0.587)	49 (47.6)	49 (47.6)	5 (4.9)	0 (0.0)	0 (0.0)
Length of time provided for solving the METAPAD gaming Puzzle was sufficient.	4.03 (0.798)	30 (29.1)	50 (48.5)	19 (18.4)	4 (3.9)	0 (0.0)
The material on the METAPAD gaming Puzzle was pertinent.	4.11 (0.670)	27 (26.2)	62 (60.2)	12 (11.7)	2 (1.9)	0 (0.0)
Remembering the enzyme and metabolite names was easier because of the METAPAD gaming Puzzle.	4.16 (0.764)	35 (34.0)	53 (51.5)	11 (10.7)	4 (3.9)	0 (0.0)
Solving METAPAD gaming Puzzle is a good review of the material covered in the lecture.	4.32 (0.564)	37 (35.9)	63 (61.2)	2 (1.9)	1 (1.0)	0 (0.0)
Extra credit should be associated with activities such as a METAPAD gaming Puzzle.	4.28 (0.678)	38 (36.9)	59 (57.3)	4 (3.9)	1 (1.0)	1 (1.0)
METAPAD gaming Puzzle is a good tool to study and revise the complex content of metabolic pathways.	4.35 (0.696)	46 (44.7)	49 (47.6)	7 (6.8)	1 (1.0)	0 (0.0)
METAPAD gaming Puzzle promoted meaningful learning instead of traditional classroom learning	4.52 (0.557)	57 (55.3)	43 (41.7)	3 (2.9)	0 (0.0)	0 (0.0)
METAPAD gaming Puzzle promotes active independent learning, student reasoning and communication skills	4.48 (0.654)	57 (55.3)	39 (37.9)	6 (5.8)	1 (1.0)	0 (0.0)

Upon analysing the correlation between age and gender independently with the perceptions towards METAPAD gaming puzzle, there was no significant difference found in students’ perceptions of METAPAD gaming puzzle among the different age groups and gender. All the students below and above 20 years of age irrespective of gender had perceived the METAPAD gaming puzzle to be an integrated and joyful learning tool. Tables 6 and 7 provide a detailed representation of the association between age and gender with perception towards the METAPAD gaming puzzle. Furthermore, on analysing the association between different learning styles and perception of the METAPAD gaming puzzle, there was a significant difference in students’ perception towards the statement, “We need to be focused to solve the METAPAD gaming Puzzle” (*p* = 0.001) among different types of learners. No other significant difference in students’ perception was identified in any of the other statements. Table 8 gives a detailed statistical analysis of the perceptions of the METAPAD gaming puzzle across different learning styles according to the VARK scale.

**Table 6:** Association of students’ perceptions of METAPAD gaming puzzle with age

Perception Survey Items	Age </= 20 N (%)	Age > 20 N (%)	*P*- value
I have enjoyed solving the METAPAD gaming Puzzles.	4.64 (0.483)	4.67 (0.492)	0.843
The clinical cases given in METAPAD gaming Puzzle were interesting	4.54 (0.523)	4.75 (0.452)	0.185
METAPAD gaming Puzzle tool stimulates my curiosity in learning the complex metabolic pathways in biochemistry.	4.58 (0.559)	4.42 (0.900)	0.727
The METAPAD gaming Puzzle tool polishes my creativity.	4.45 (0.601)	4.33 (0.778)	0.703
The METAPAD gaming Puzzle tool enhances my problem-solving ability.	4.55 (0.563)	4.50 (0.522)	0.668
The METAPAD gaming Puzzles helped in improving my critical thinking ability	4.51 (0.524)	4.50 (0.674)	0.841
We need to be focused to solve the METAPAD gaming Puzzle	4.63 (0.509)	4.50 (0.674)	0.523
I am able to recall the topics while solving the METAPAD gaming Puzzle.	4.21 (0.606)	4.08 (0.900)	0.838
The METAPAD gaming Puzzles can be used to evaluate a student too.	3.84 (0.910)	3.67 (0.888)	0.523
METAPAD gaming Puzzle can increase interaction among the students.	4.69 (0.464)	4.75 (0.452)	0.684
The METAPAD gaming Puzzle enhanced my learning.	4.44 (0.581)	4.50 (0.522)	0.805
I have enjoyed classmate interaction and reviewing the content of metabolic pathways while solving the METAPAD gaming Puzzle.	4.62 (0.511)	4.50 (0.674)	0.644
METAPAD gaming Puzzle oriented us to the topics that we should focus on.	4.43 (0.580)	4.42 (0.669)	0.972
Length of time provided for solving the METAPAD gaming Puzzle was sufficient.	4.08 (0.763)	3.67 (0.985)	0.118
The material on the METAPAD gaming Puzzle was pertinent.	4.13 (0.618)	3.92 (0.996)	0.517
Remembering the enzyme and metabolite names was easier because of the METAPAD gaming Puzzle.	4.12 (0.786)	4.42 (0.515)	0.257
Solving METAPAD gaming Puzzle is a good review of the material covered in the lecture.	4.32 (0.575)	4.33 (0.492)	0.961
Extra credit should be associated with activities such as a METAPAD gaming Puzzle.	4.29 (0.688)	4.25 (0.622)	0.728
METAPAD gaming Puzzle is a good tool to study and revise the complex content of metabolic pathways.	4.34 (0.703)	4.42 (0.669)	0.739
METAPAD gaming Puzzle promoted meaningful learning instead of traditional classroom learning	4.55 (0.543)	4.33 (0.651)	0.252
METAPAD gaming Puzzle promotes active independent learning, student reasoning and communication skills	4.49 (0.639)	4.33 (0.778)	0.506

**Table 7:** Association of students’ perceptions of METAPAD gaming puzzle with gender

Perception Survey Items	Gender Male Mean (Sd)	Gender Female Mean (Sd)	*P*-Value
I have enjoyed solving the METAPAD gaming Puzzles.	4.57 (0.502)	4.68 (0.471)	0.295
The clinical cases given in METAPAD gaming Puzzle were interesting	4.60 (0.497)	4.54 (0.531)	0.650
METAPAD gaming Puzzle tool stimulates my curiosity in learning the complex metabolic pathways in biochemistry.	4.49 (0.781)	4.60 (0.493)	0.848
The METAPAD gaming Puzzle tool polishes my creativity.	4.46 (0.701)	4.43 (0.581)	0.582
The METAPAD gaming Puzzle tool enhances my problem-solving ability.	4.51 (0.658)	4.56 (0.500)	1.000
The METAPAD gaming Puzzles helped in improving my critical thinking ability	4.46 (0.611)	4.53 (0.503)	0.690
We need to be focused to solve the METAPAD gaming Puzzle	4.54 (0.505)	4.65 (0.540)	0.236
I am able to recall the topics while solving the METAPAD gaming Puzzle.	4.20 (0.719)	4.19 (0.605)	0.757
The METAPAD gaming Puzzles can be used to evaluate a student too.	3.69 (0.963)	3.88 (0.873)	0.386
METAPAD gaming Puzzle can increase interaction among the students.	4.74 (0.443)	4.68 (0.471)	0.489
The METAPAD gaming Puzzle enhanced my learning.	4.40 (0.695)	4.47 (0.503)	0.886
I have enjoyed classmate interaction and reviewing the content of metabolic pathways while solving the METAPAD gaming Puzzle.	4.54 (0.611)	4.63 (0.486)	0.601
METAPAD gaming Puzzle oriented us to the topics that we should focus on.	4.57 (0.558)	4.35 (0.593)	0.068
Length of time provided for solving the METAPAD gaming Puzzle was sufficient.	4.03 (0.857)	4.03 (0.772)	0.889
The material on the METAPAD gaming Puzzle was pertinent.	4.00 (0.804)	4.16 (0.589)	0.425
Remembering the enzyme and metabolite names was easier because of the METAPAD gaming Puzzle.	4.09 (0.781)	4.19 (0.758)	0.514
Solving METAPAD gaming Puzzle is a good review of the material covered in the lecture.	4.31 (0.676)	4.32 (0.502)	0.735
Extra credit should be associated with activities such as a METAPAD gaming Puzzle.	4.26 (0.817)	4.29 (0.600)	0.867
METAPAD gaming Puzzle is a good tool to study and revise the complex content of metabolic pathways.	4.34 (0.838)	4.35 (0.617)	0.672
METAPAD gaming Puzzle promoted meaningful learning instead of traditional classroom learning	4.54 (0.611)	4.51 (0.532)	0.637
METAPAD gaming Puzzle promotes active independent learning, student reasoning and communication skills	4.46 (0.780)	4.49 (0.586)	0.755

**Table 8:** Students’ Perceptions of METPAD gaming Puzzle across Preferred Learning Styles

Perception Survey Items	Visual (*N* = 11) Mean (SD)	Aural (*N* = 7) Mean (SD)	Read/write (*N* = 4) Mean (SD)	Kinesthetic *(N* = 30) Mean (SD)	Multimodal (*N* = 51) Mean (SD)	*P*- Value
I have enjoyed solving the METAPAD gaming Puzzles.	4.55 (0.522)	4.57 (0.535)	4.75 (0.500)	4.60 (0.498)	4.69 (0.469)	0.836
The clinical cases given in METAPAD gaming Puzzle were interesting	4.55 (0.522)	4.14 (0.690)	4.50 (0.577)	4.53 (0.507)	4.65 (0.483)	0.313
METAPAD gaming Puzzle tool stimulates my curiosity in learning the complex metabolic pathways in biochemistry.	4.09 (0.944)	4.57 (0.535)	4.75 (0.500)	4.63 (0.490)	4.61 (0.568)	0.320
The METAPAD gaming Puzzle tool polishes my creativity.	4.09 (0.539)	4.00 (0.816)	4.50 (0.577)	4.53 (0.571)	4.51 (0.612)	0.092
The METAPAD gaming Puzzle tool enhances my problem-solving ability.	4.55 (0.522)	4.29 (0.488)	4.50 (0.577)	4.47 (0.504)	4.57 (0.608)	0.655
The METAPAD gaming Puzzles helped in improving my critical thinking ability	4.27 (0.786)	4.43 (0.535)	4.50 (0.577)	4.50 (0.509)	4.57 (0.500)	0.793
We need to be focused to solve the METAPAD gaming Puzzle	4.36 (0.505)	3.86 (0.378)	4.75 (0.500)	4.77 (0.430)	4.67 (0.516)	**0.001**
I am able to recall the topics while solving the METAPAD gaming Puzzle.	3.91 (0.831)	3.71 (0.756)	4.50 (0.577)	4.30 (0.535)	4.24 (0.619)	0.160
The METAPAD gaming Puzzles can be used to evaluate a student too.	3.45 (0.820)	3.00 (0.816)	4.25 (0.957)	4.00 (0.695)	3.86 (0.980)	0.051
METAPAD gaming Puzzle can increase interaction among the students.	4.82 (0.405)	4.43 (0.535)	4.75 (0.500)	4.83 (0.379)	4.63 (0.488)	0.137
The METAPAD gaming Puzzle enhanced my learning.	4.55 (0.522)	3.71 (0.756)	4.75 (0.500)	4.50 (0.509)	4.47 (0.542)	0.052
I have enjoyed classmate interaction and reviewing the content of metabolic pathways while solving the METAPAD gaming Puzzle.	4.45 (0.820)	4.43 (0.535)	4.50 (0.577)	4.63 (0.490)	4.65 (0.483)	0.820
METAPAD gaming Puzzle oriented us to the topics that we should focus on.	4.45 (0.522)	4.00 (0.577)	4.75 (0.500)	4.40 (0.621)	4.47 (0.578)	0.277
Length of time provided for solving the METAPAD gaming Puzzle was sufficient.	4.00 (0.775)	3.86 (0.378)	3.75 (0.957)	4.03 (0.669)	4.08 (0.913)	0.769
The material on the METAPAD gaming Puzzle was pertinent.	3.82 (0.874)	3.57 (0.535)	4.25 (0.500)	4.13 (0.507)	4.22 (0.702)	0.082
Remembering the enzyme and metabolite names was easier because of the METAPAD gaming Puzzle.	4.36 (0.674)	3.71 (0.756)	4.50 (0.577)	4.10 (0.803)	4.18 (0.767)	0.407
Solving METAPAD gaming Puzzle is a good review of the material covered in the lecture.	4.27 (0.647)	4.29 (0.488)	4.50 (0.577)	4.30 (0.466)	4.33 (0.622)	0.932
Extra credit should be associated with activities such as a METAPAD gaming Puzzle.	4.09 (0.539)	4.00 (1.414)	4.00 (0.816)	4.43 (0.504)	4.29 (0.642)	0.469
METAPAD gaming Puzzle is a good tool to study and revise the complex content of metabolic pathways.	4.00 (0.775)	4.29 (0.488)	4.75 (0.500)	4.50 (0.509)	4.31 (0.787)	0.226
METAPAD gaming Puzzle promoted meaningful learning instead of traditional classroom learning	4.36 (0.674)	4.57 (0.535)	4.50 (0.577)	4.63 (0.490)	4.49 (0.579)	0.767
METAPAD gaming Puzzle promotes active independent learning, student reasoning and communication skills	4.27 (0.786)	4.57 (0.535)	4.75 (0.500)	4.50 (0.572)	4.47 (0.703)	0.806

For the first open ended question, “do you wish to make any additional comments?”, 29 students responded through the survey. In addition to that, personal interviews were conducted team-wise to collect the feedback of the students regarding METAPAD gaming puzzle and their responses were recorded. Consent was obtained from the respondents to use the recordings anonymously for research purpose.

### Thematic analysis of students’ feedback

After the analysis of students’ comments and recordings through the perception survey and personal interviews, all the feedback was categorised into 12 common themes. The themes were then verified to ensure that they represent the original meaning after feedback. All the feedback received from the students was carefully assigned to the respective themes.

**Fig. 3:**
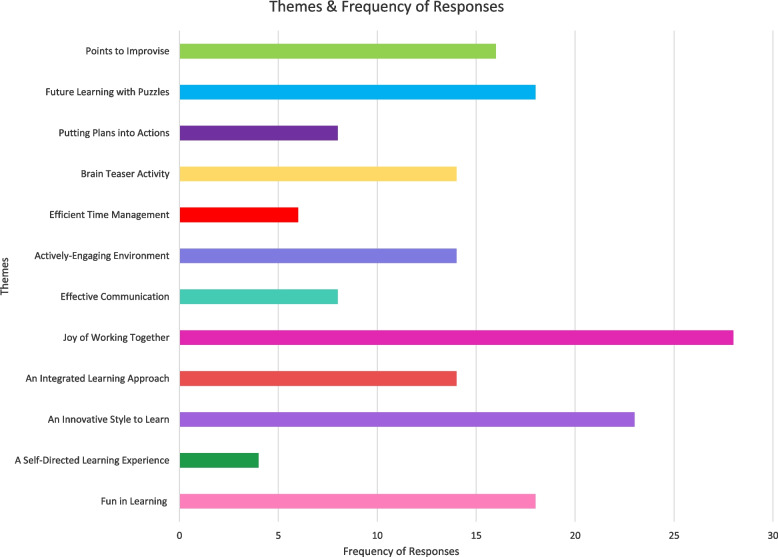
Frequency of responses included under each theme

Based on the students comments, the assigned themes were (1) Fun in Learning, (2) A Self-Directed Learning Experience, (3) An Innovative Style to Learn, (4) An Integrated Learning Approach, (5) Joy of Working Together, (6) Effective Communication, (7) Actively-Engaging Environment, (8) Efficient Time Management, (9) Brain Teaser Activity, (10) Putting Plans into Actions, 11) Future Learning with Puzzles and 12) Points to Improvise. Themes and their frequency in the feedback are shown in Fig. 3 and explained in Tables 9, 10 and 11. A total of 171 responses with grouped under 12 themes. Few representative comments are enumerated below

**Table 9:** Thematic analysis of students’ free / open comments on METAPAD gaming puzzle activity − 1

**Fun in learning**	**A self-directed learning experience**
*“It was innovative and fun”* *“Normal way of thinking is hard but this made it fun”* *“It was fun, we had a good time”* *“It was bit hard but it was very fun to work in teams”* *“It was very interesting fun activity, expected something else but it was a totally different experience”* *“It’s fine it was easy to learn all pathways related to diabetes”* *“It was interesting and fun activity to do and it was a unique way of learning metabolic pathways”* *“It was very nice enjoyed it thoroughly”* *“It was difficult in level 2 but it was totally nice we had lots of fun”* *“ good experience”* *“I feel this was very interesting and help the students in many ways”* *“It is a fun filled learning session”* *“It was fun and creative”* *“It was fun and learning was easy* *“Informative and useful program and had so much fun in learning while solving the puzzle”* *“Loved the session”* *“METAPAD 2022 was awesome, really enjoyed it”* *“Really fun and enthusiastic session”*	*“It’s like a trial and error method where we try to match the pathway and when you realise it is not correct you change by this we are learning it on our own”* *“It’s on us and we do all the stuff”* *“It’s different from traditional methods as here we ourselves are engaged in this learning process”* *“We indulge ourselves into this every time and every moment so even if academics go in this way it will be better, it is an innovative and self-directed learning process”*
**An innovate style to learn**	**An integrated learning approach**
*“Very different concept from what we usually do here in college, it really made our mind work alot because we had to do the puzzle”* *“It was both educational as well as fun activity, so it was a combination, we didn’t feel the stress like we are learning something complex”* *“We learn something new that we did not even know earlier, it is very innovative”* *“METAPAD Is an innovative style of learning”* *“It’s a new idea a nice to decode metabolic pathways and inclusion of clinical case was very nice idea and increased our enthusiasm and energy and made us more creative and active”* *“This METAPAD Was so different from actual learning and enjoyable ”* *“Very innovative way of helping the students”* *“Knowledge gaining session”* *“METAPAD- stimulates creativity among us”* *“It’s more kinaesthetic type of learning where we involve ourselves to learn”* *“Helpful in learning the pathways in a new way rather than writing it, it increased our memory power”* *“It has helped me to learn more about biochemistry and how to make it interesting”* *“It had helped me to focus on minute things about the pathways”* *“It didn’t make us feel bored and it was very interesting that we could learn a pathway completely without any ups and downs which we face in traditional classroom”* *“If we study metabolism individually it will take around 30 minutes but while solving it as a puzzle, it is quite interesting and takes shorter span of time”* *“In classes we simply sit and listen that time we sleep too but here we actually have to work”* *“We were able to engage ourselves in learning instead of listening to lectures and it helped us to understand the concepts”* *“Different from normal way of understanding metabolic pathways”* *“It made us more eager to learn”* *“It was interesting and style was innovative at the same time it was little bit tense”* *“It was well organised and well planned”* *“If can be integrated with real life lectures it will be interesting to learn”* *“I would love to learn more concepts through this way of metabolic gaming puzzle. This is really a useful learning method which requires only a short span of time and contents can be remembered forever”*	*“We study synthesis separately and degradation separately but when solving this we had to integrate everything”* *“We learnt a new way to study, previously we learned it in step by step manner now we can combine and relate the pathways”* *“We could learn all the pathways in a different and fun way, when we assemble things only we understand where this step comes and where are the step comes”* *“You get the knowledge about the whole cycle instead of just the steps”* *“We thought it was a linear pathway but it was a circle, a combination of both lysis and genesis so we had to work more and integrate both the cycles”* *“I could relate the pathways that I have learnt in a different way”* *“Before when I used to study biochemistry, I was not able to connect the cycle and study but once we started solving this we were trying to connect all the pathways”* *“It was a 2 way learning, simultaneously we were learning the pathway and theory behind it also”* *“I was able to recall all the enzymes names, it made me think about the whole cycle in a different way”* *“It helped me to learn the concepts very well, I was able to learn different compounds and different enzymes names thoroughly from this”* *“We should not solve just by learning we should also use our skills”* *“Puzzle learning is enjoyable and easy to remember and helps to quickly identifying what you have missed”* *“Solving these puzzles not only helped us in revising but also getting to know the”* *“It increases the orientation of learning perspectives in the learner”*

**Table 10:** Thematic analysis of students’ Free / open comments on METAPAD gaming puzzle activity – 2

**Joy of working together**	**Effective communication**
*“While doing it in teams it was very interesting we can learn new things”* *“Best part was team effort, everybody was united to finish it as quickly as possible”* *“It’s new experience for us like going through a new face where we could mingle with a lot of people with different mentalities”* *“It’s all about teamwork”* *“It was very challenging for us as a group to find all the pieces and put them together”* *“It was fun to do it with our classmates but it was hard in the second round compared to the first round but we all work together”* *“We got to know each other better and finally we could do everything as a group”* *“The coordination between the teams was good”* *“It helped us to work together in teams and it is a nice initiative by the department”* *“We felt that we were able to spend more educational time with our friends”* *“It’s more easy while working in teams I can easily understand the concepts in depth”* *“It was helpful learning with friends and now glycogen metabolism means I remember fully”* *“Instead of constant learning from book we were working as a team to decode the pathway which was really interesting”* *“Team skills is very important”* *“If one person doesn’t know the answer the other person explained so it was very easy to put together and see as a bigger picture”* *“There was good coordination among us”* *“It helped us work in a team, generally while solving puzzles we do it individually but this was like a teamwork”* *“We should have a clear idea about the pathway and we should work in teams”* *“We cannot work as teams in class but here we were able to communicate and work as team”* *“Even when I didn’t know the pathway my friends thought me saying you have to do this you have to do that so it was fun learning”* *“Understanding and patience is very important while working in teams”* *“We learn how to work in a team each one will have a different view and we learn how to put together every single view”* *“It was nice to work with others* *I learnt that it’s always better to study in a team and perform in a team”* *“Through this, we have learnt team learning in short time we need to correlate the metabolism”* *“We cooperative and friends, so we sort it together”* *“Teams is more efficient than working alone”* *“We were helping each other and we found ourselves on the finish line”* *“Even if we were talking for the first time it was easy for us to communicate in this game”*	*“Interesting and we had interaction with all the team members”* *“Better communication”* *“I think we have acquired the ability to tolerate each other and have patience and communicate better”* *“It made us more tense but we learnt new things and interaction was quite useful for us”* *“We learnt to interact, if one part of the puzzle is missing we ask each other and communicate well”* *“We were always communicating with each other while we had any problems”* *“It is easy when we communicate which is very important in puzzle solving in teams”* *“It will increase the communication skills and interaction between students”*
**Actively- engaging environment**	**Efficient time management**
*“Lectures are quite boring but if it is more like puzzles more people will engage”* *“It was very enthusiastic, seeing others do we also feel enthusiastic to solve the puzzle”* *“We concentrated more on what we did and the other teams were also really competitive”* *“The game was so intense we all are competitive”* *“We didn’t have time to look at other teams we were rushing to do our own puzzle in the most correct and quickest manner”* *“It was an energetic atmosphere”* *“Very competitive”* *“It was chaotic”* *“Friendly but competitive”* *“Environment was a healthy competition”* *“Energetic”* *“The competition made it good”* *“Good and coordinating”* *“Everybody was supporting each other”*	*“Time management is important”* *“Our main area of focus was to complete it as early as possible” “Finishing the puzzle was her main area of focus”* *“To complete it fully on time bazar main goal”* *“We were clear with the topics and we knew the cycle well so it was very useful to do it as fast as we can”* *“Main focus was time to complete it first by solving it without errors”*

**Table 11:** Thematic analysis of students’ free / open comments on METAPAD gaming puzzle activity – 3

**Brain teaser activity**	**Putting plans into actions**
*“It was training the brain and physical effort was also required”* *“it activated all our brain cells to solve the puzzle”* *“It was very interesting, first round was very easy but when we went to the second round we found how difficult it was”* *“We have to know the concept and steps to arrange the person”* *“It has changed our way of thinking”* *“We have acquired problem solving skills”* *“It has made us critically think about metabolic pathways”* *“Normally we used to see the book and learn something but here we used our creativity to match all those pathways so we remember it more accurately than normal reading”* *“Creative thinking was boosted”* *“Through case based learning it was very interesting and it improve our critical thinking”* *“It was very challenging”* *“The second level was very hard, we better not able to finish it but we were able to learn”* *“It was good somewhat confusing but we can learn with keys”* *“I feel this is as a competitive method of learning and is much helpful in stimulating the brain to understand and memorise the concepts”*	*“We all know the pathway while learning for example but when it comes to appoint of competition we had to do it at the fastest time”* *“Main area of focus boss where to put which piece”* *“To go with the flow”* *“We arranged all the words but merging them was difficult”* *“We were able to revise what we have studied earlier and also we were able to find the missed out points while we were studying”* *“While solving the puzzle, we realised that we have missed learning these points and we were able to recall those”* *“Focusing on the empty areas to arrange the puzzle”* *“It’s like an easier form of practicals, it’s like you are breaking down everything together and doing it rather than just seeing it on the board and in the book you are putting it into practice”*
**Future learning with puzzles**	**Points to improvise**
*“Amino acid metabolism”* *“Cholesterol metabolism because it has feedback mechanism”* *“Molecular biology”* *“Proteins, urea cycle, vitamins importantly lipid storage diseases”* *“Molecular biology- it is very dry to have a lecture class having puzzles in these topics would be helpful”* *“All pathways”* *“Whole biochemistry can be learned with puzzles”* *“It’s just not about the metabolic pathways, most of the things if we learn like this it will be better”* *“For visual, kinaesthetic and multimodal learners it would be better to learn it in a gaming way”* *“This time we did the pathways maybe next time we could do the disorders related to every pathway that will be fun”* *“Like phenylketonuria heart nerves disease they can show symptoms as a chart and we can arrange them”* *“All the metabolic pathways”* *“Every topic in biochemistry”* *“We can also learn shorter pathways like this instead of only the major”* *“Vitamins and minerals matching with RDA values or something like that”* *“It is done for all important metabolism it will be useful”* *“Please keep METAPAD instead of revision classes it will help us learn and remember this important points and help us to reason out better”* *“Would like to do it for learning all the topics but would like to have shorter session so that more topics can be covered in a shorter time”*	*“While solving the puzzles there were many blank spaces, it was difficult to arrange all that and make it a perfect rectangle”* *“Next time there can be puzzles without lot of blank cards so that we won’t get confused”* *“Puzzles with pictures can be interesting”* *“Puzzle size can be bigger”* *“The puzzles can be broken with complete words instead of breaking the words, then it would be much easier to correlate with what we learnt and it would take lesser time”*

*“This is your and the biochemistry's brain child. Thank you for giving us such a different, unique, mind blowing and fun opportunity. This has helped us develop team involvement and interaction which has increased our knowledge of each other and learnt different ways of working with each other. So thank you once again.”*

*“Initially we were new to the team so we don't know each other very much, so it started off a bit rough and then later on we communicated with each other well and we decided and discussed how to solve the puzzle as a team and we all became friends now and we ended up winning”*

*“Learning by solving these kind of puzzles made it easier to remember things the cycles are hard to memorise but even harder to keep it in mind retention is very low but these puzzles helped in memorising, I think if puzzles were implemented in all the chapters that we learn it would be helpful even more”*

**“** *It was very interesting, it made me recall the pathways that I have learnt and interlink the pathways, eg: glycogen metabolism I learnt both the pathways separately but when they gave together I learnt how to incorporate both ”*

### Factor analysis of the perceptions items

The questionnaire used to evaluate students’ perception towards the METAPAD gaming puzzle as an effective learning tool was divided into 4 domains: (i) METAPAD Game Materials (5 items), (ii) Brainstorming with METAPAD (4 items), (iii) METAPAD – earning Experiences (6 items) and (iv) Advantages of METAPAD (6 items). Factor analysis for the METAPAD gaming puzzle perceptions is represented in Table 12.

**Table 12:** Factor analysis of perceptions towards METAPAD gaming puzzle

S.No	Questions	Component 1	Component 2
	Domain 1: METAPAD GAME MATERIALS		
1.	I have enjoyed solving the METAPAD gaming Puzzles.	0.787	0.100
2.	**The clinical cases given in METAPAD gaming Puzzles were interesting.**	**0.854**	0.112
3.	METAPAD gaming Puzzle oriented us to the topics that we should focus on.	0.592	0.470
4.	**Length of time provided for solving the METAPAD gaming Puzzle was sufficient.**	0.047	**0.878**
5.	The material on the METAPAD gaming Puzzle was pertinent.	0.244	0.803
	**Domain 2: BRAINSTORMING WITH METAPAD**		
6.	**METAPAD gaming Puzzle tool stimulates my curiosity in learning the complex metabolic pathways in biochemistry.**	**0.844**	-
7.	The METAPAD gaming Puzzle tool polishes my creativity.	0.722	-
8.	The METAPAD gaming Puzzle tool enhances my problem-solving ability.	0.769	-
9.	The METAPAD gaming Puzzles helped in improving your critical thinking ability?	0.775	-
	**Domain 3: METAPAD – LEARNING EXPERIENCES**		
10.	We need to be focused to solve the METAPAD gaming Puzzle.	0.593	-
11.	I am able to recall the topics while solving the METAPAD gaming Puzzle.	0.628	-
12.	The METAPAD gaming Puzzles can be used to evaluate a student too.	0.702	-
13.	METAPAD gaming Puzzle can increase interaction among the students.	0.628	-
14.	**The METAPAD gaming Puzzle enhanced my learning.**	**0.758**	-
15.	I have enjoyed classmate interaction and reviewing the content of metabolic pathways while solving the METAPAD gaming Puzzle.	0.554	-
	**Domain 4: ADVANTAGES OF METAPAD**		
16.	Remembering the enzyme and metabolite names was easier because of the METAPAD gaming Puzzle.	0.668	-
17.	Solving METAPAD gaming Puzzle is a good review of the material covered in the lecture.	0.830	-
18.	Extra credit should be associated with activities such as a METAPAD gaming Puzzle.	0.643	-
19.	**METAPAD gaming Puzzle is a good tool to study and revise the complex content of metabolic pathways.**	**0.839**	-
20.	METAPAD gaming Puzzle promoted meaningful learning instead of traditional classroom learning	0.815	-
21.	METAPAD gaming Puzzle promotes active independent learning, student reasoning and communication skills	0.731	-

### Domain 1: METAPAD game materials

**Fig. 4:**
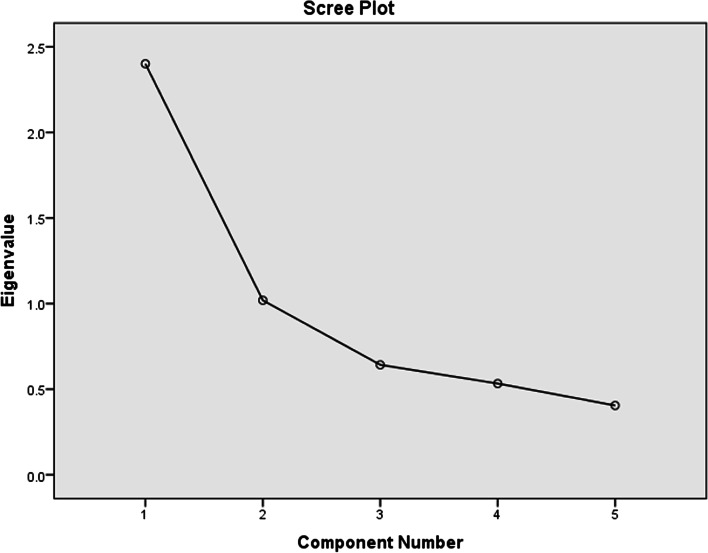
Scree plot for domain 1

Considering the first domain that evaluated students’ perceptions towards the METAPAD game materials, An Eigenvalue of more than 1 was obtained for 2 components. The scree plot for the same is represented in Fig. 4. As there are 2 components, the rotational component matrix reveals, “The clinical cases given in METAPAD gaming Puzzles were interesting” and “Length of time provided for solving the METAPAD gaming Puzzle was sufficient” as the most influencing factors with component values of 0.054 and 0.878.

### Domain 2: brainstorming with METAPAD

**Fig. 5:**
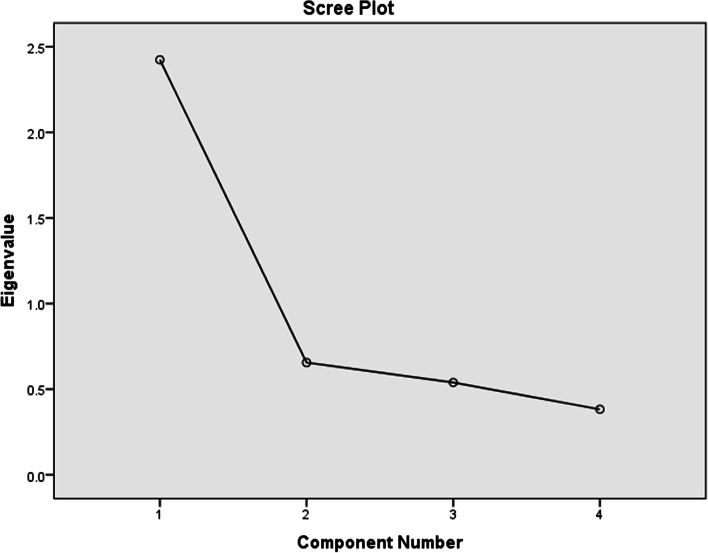
Scree plot for domain 2

Secondly, upon analyzing the most influencing factors in Domain 2 that included evaluating brainstorming with METAPAD, the scree plot revealed one component with an Eigenvalue of more than one. Thus, the component matrix (without rotation) indicated that “METAPAD gaming Puzzle tool stimulates my curiosity in learning the complex metabolic pathways in biochemistry” was the most influencing with a component value of 0.844. Figure 5 represents the scree plot for Domain 2.

### Domain 3: METAPAD – learning experiences

**Fig. 6:**
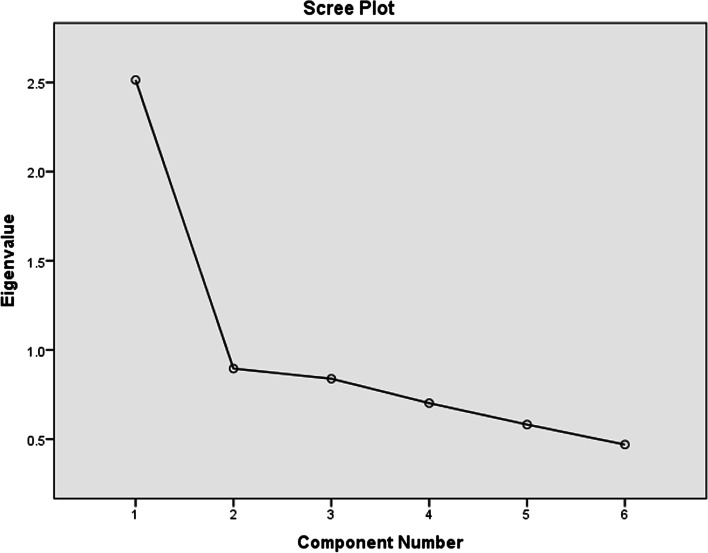
Scree plot for domain 3

Domain 3 included students’ perceptions regarding their learning experiences with METAPAD gaming puzzle. Figure 6 shows the scree plot with one component having an Eigenvalue of more than 1. Thus, without rotation, the component matrix indicated that “The METAPAD gaming Puzzle enhanced my learning” is the most influencing in the domain with a component value of 0.758.

### Domain 4: advantages of METAPAD

**Fig. 7:**
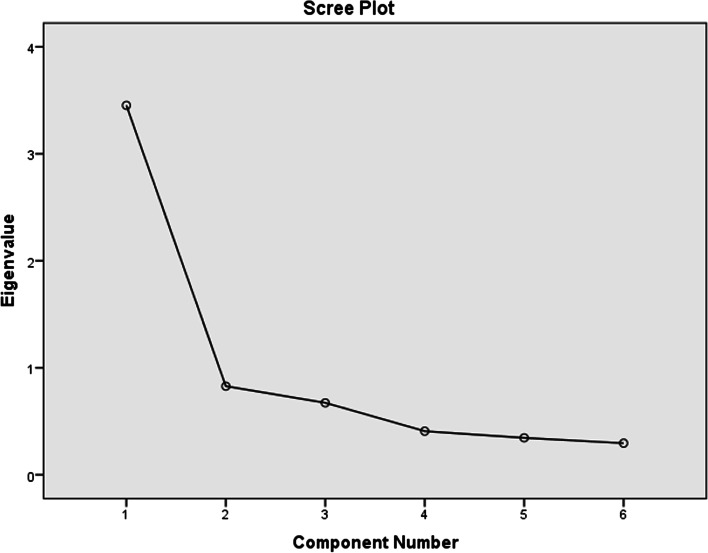
Scree plot for domain 4

Finally, the last domain included the perceptions of students in regard to the advantages of METAPAD. An Eigenvalue of more than 1 was obtained for only one component as represented in Fig. 7. Thus, the component matrix without rotation revealed that “METAPAD gaming Puzzle is a good tool to study and revise the complex content of metabolic pathways” is the most influencing with a component value of 0.839.

## Discussion

The complex metabolic pathways and intricate molecular mechanisms in Biochemistry is vital for undergraduate medical students to understand and integrate with clinical scenarios. These topics are usually introduced in the first year of medical school along with Anatomy and Physiology metabolic pathways being distinct from other subjects require deep understanding and clarity to correlate it with clinical conditions. But most of the students face difficulty in learning the complicate pathways and retaining the information during exams which causes a misconception that Biochemistry is very hard to learn. Non-visualization of molecular mechanisms, remembering newer enzymes and metabolite names and understanding complex cycles make it hard for students to comprehend biochemical concepts in a conventional didactic mode of learning.

Thus, to facilitate the students learning process in a fun-filled and engaging manner, the game-based education strategy was introduced to enhance students’ participation and interaction to promote optimum learning. To the best of our knowledge, this is the first study that has been conducted in biochemistry for undergraduate medical students to evaluate students perceptions of introducing the component of game, case-based learning and team-based learning in a more self-directed way and the association between their preferred learning styles.

**Fig. 8:**
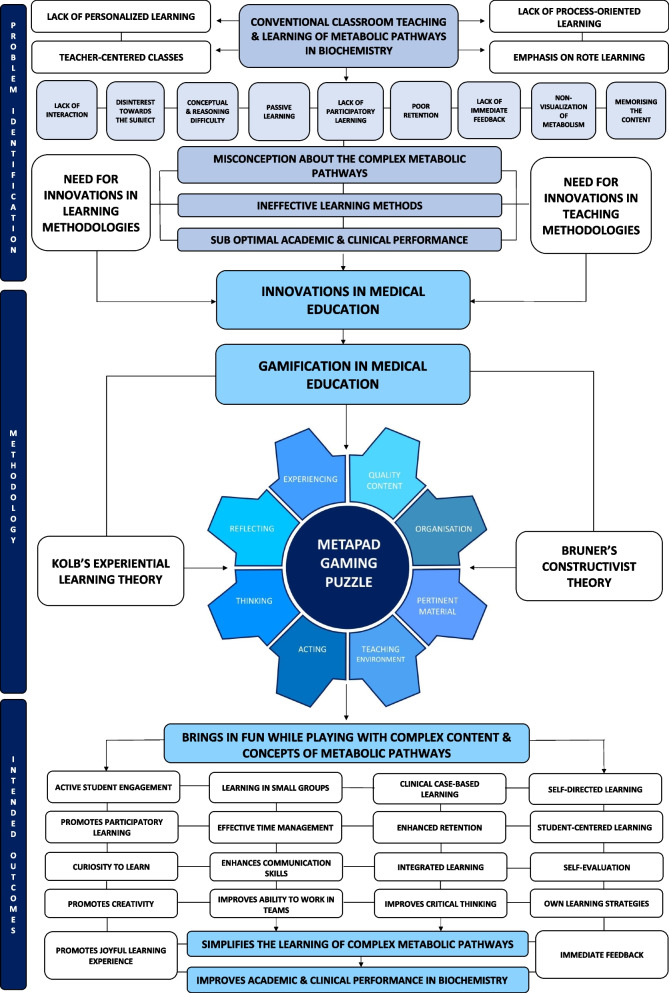
Conceptual Framework of METAPAD (METAbolic PAthways Decoded)

In this study, students’ perception towards game based learning is highly optimistic. On the whole, most of the students perceived that they enjoyed solving the METAPAD gaming puzzle and it has facilitated their learning by working in teams. Majority of the students agreed that the clinical case given to solve and puzzle-based approach enhance their understanding and clinical interpretation skills by orienting them to the topic, helping then focus and integrate what they have learnt and served as a good review of material covered in the lectures. These results are compatible with other studies that have been published in regards to introducing game based learning [6, 15, 16]. A Prochazkova K et al. reported that learning by playing will motivate the students towards continuous learning and is an important tool in teaching biochemistry [17]. The findings of our study align with previous research that has investigated the effectiveness of puzzles and games in promoting constructive learning. Introducing puzzle-based games in the learning environment stimulates students to actively participate in lively discussions with their team members. These discussions foster collaborative learning experiences, allowing students to exchange ideas, clarify concepts, and deepen their understanding of the subject matter and it can be used as an effective educational alternative in biochemistry to enhance knowledge and obtain better academic outcomes [18–22] (Fig. 8).

In the present study there was no significant relationship between gender and perception towards METAPAD gaming puzzle. While there was a significant difference reported among males and females in the study that used the same survey instrument by *Ghada Bawazeer et al.* [14], towards the question that assessed the perceptions on the length of time provided for solving puzzles. Male students did not show much satisfaction towards the time allocated to solve the puzzle in their study. Whereas, our study and other studies that evaluated the agreement on allocated time to solve a puzzle did not show any conflict among the participants [5, 23–26]. This is supported by Demirören M et al., scientific report on gender differences in self-regulated learning That states there exists no significant relationship between males and females while learning in groups, interacting and their thinking ability [27].

The same study evaluating the association between different learning styles and perceptions of crossword puzzles in pharmacology reported no significant difference among the two [14]. In contrast, the current study’s results highlight the importance of considering the match between learning styles and instructional approaches when incorporating innovative educational tools like the METAPAD gaming puzzle. The significant difference suggests that learners with certain learning styles may find the puzzle more engaging and requiring greater focus, potentially enhancing their learning experience and outcomes. In medical education, the predominant learning style is of multimodal type followed by kinaesthetic while other the occurrence of unimodal styles like visual, auditory, read and write are relatively low [28–32]. However gender and previous academic performance was found to have no influence over the preferred learning styles [33]. the predominant learning style in our study is multimodal (49.5%) and kinaesthetic (29.1%). According to VARK modalities, visual learners prefer more of diagrams, maps, graphs and flow charts as a medium for acquiring knowledge. auditory learners grasp information through talking, explaining, listening and discussing the content. read and write type of learners on the other hand, are able to learn effectively when they write or read the content and study. These type of learners often surf the internet for explanations. While kinaesthetic learners use experience and practice as a way of learning new things. They strongly prefer ‘learning by doing’ that keeps them engaged and helps them to retain information for long time. Lastly, the multimodal learners are a combination of all the VARK styles of learning and prefer more than one method to learn [13]. However, regardless of the type of learning styles, games create a more self-evaluated and effective environment to learn that is preferred by all types of learners [34]. In our study, learning style preferences did not affect the overall acceptance of the METAPAD gaming puzzle as an effective learning tool. However, the only significant result obtained was in relation to the requirement to focus more while solving the METAPAD gaming puzzle among different types of learners. This suggests that learners with varying preferences and approaches to learning may have different levels of engagement and focus when solving puzzle-based games. But the majority of the perceptions towards METAPAD gaming puzzle as an effective learning tool were similar among different types of learners indicating that learning styles did not stand as a barrier to the acceptance of our innovation.

The feedback of students on the METAPAD gaming puzzle was also analysed and categorised them under 12 teams. After 171 responses, majority of them were included under the theme ‘Joy of working together’ (*n* = 28), ‘An innovative style to learn’ (*n* = 23), ‘Fun in learning’ (*n* = 18), and ‘Future learning with puzzles’ (*n* = 18). This shows that students enjoy learning in groups which enhances the interaction among them and improve their relationship with peers. Students’ preference over puzzle and game based learning to traditional classroom teaching for all topics in biochemistry shows that students are always interested in innovative learning styles that are fun-filled and engaging. This will certainly motivate them to learn and promote lifelong learning in Biochemistry.

Furthermore, the factor analysis report provided the most influencing factors in each of the four domains included. Considering domain 1, factor analysis revealed that the most significant factors influencing participant engagement in METAPAD gaming Puzzles were the interesting nature of the clinical cases and the sufficient time provided for solving them. These factors were found to have a strong positive correlation with participants’ motivation, critical thinking, and overall satisfaction with the puzzles. Thus, ensuring a continuous supply of intriguing cases and reasonable time limits is vital for maximizing engagement and optimizing problem-solving outcomes in the gaming platform. In domain 2, the factor analysis report indicated that the most influential factor among participants is the stimulation of curiosity in learning complex metabolic pathways in biochemistry through the METAPAD gaming Puzzle tool. This finding suggests that the gaming tool effectively promotes interest and engagement in the subject matter, encouraging active learning and fostering a deeper understanding of biochemistry among students. Similarly, the factor analysis of Domain 3 indicated the enhancement of learning through the METAPAD gaming Puzzle as the most influential factor. This indicates that the gaming tool significantly contributes to participants’ educational experience, suggesting that it is an effective and valuable platform for promoting knowledge acquisition and understanding. Finally, in domain 4, the factor analysis highlighted that the most influential factor is the effectiveness of the METAPAD gaming Puzzle as a tool for studying and revising complex metabolic pathways. This finding has a good educational impact suggesting that the gaming tool is highly regarded by participants for its ability to facilitate learning and review of intricate biochemistry content, making it a valuable resource for educational purposes.

### Implications of the study

From the results of our study, it is evident that the METAPAD gaming puzzle has significant implications for medical education. METAPAD gaming puzzle fosters a more engaging and interactive learning environment and enhances learning in a self-directed manner by promoting critical thinking, problem-solving skills and active participation. This is a holistic fun-filled activity that stimulates curiosity, enhances creativity, and improves students’ ability to recall and apply knowledge. Engaging students in active learning of complex metabolic pathways in biochemistry is a challenging task for medical educators. But through the results of our study, the effectiveness of games such as METAPAD in engaging students in active learning and improving knowledge retention is now evidence-informed. Also, age, gender or learning styles did not serve as a barrier to the overall acceptance and adoption of the METAPAD gaming puzzle. This unanimous support towards the introduction of games in the learning environment signifies that the implementation of the METAPAD gaming puzzle can be a paradigm shift in learning biochemistry where students experience brainstorming sessions, encouraging them to collaborate, integrate, evaluate and effectively learn the concepts as well as retain them for longer periods. Although consistent with the overall findings of other studies in stating that games enhance learning and collaboration, our study is significant in terms of identifying that needs of learners change with different types of games. For solving puzzle-based games like METAPAD, learners required more focus. Thus, understanding the relationship between the needs of diverse learners and different types of games can help medical educators tailor their instructional methods, ensuring a more personalized and effective learning experience for all students.

### Strengths of the study

The strength of our study is that the METAPAD gaming puzzle combines both Constructivist and Experiential Learning Theories to deliver an efficient teaching-learning approach to ease the learning of biochemistry. Our innovation encompasses the component of ‘fun’ and ‘teamwork’ through puzzle games conducted in small groups. It is also the first of its kind to incorporate case-based learning into game-based learning in order to facilitate the best learning experience. Our study also analysed the perceptions of students both quantitatively and qualitatively using exhaustive data collected from them. In addition to this, factor analysis for the questions included in the perception survey was done to identify the most influential factor in each domain. Thus, this study gives an explicit outline of using game-based innovations to learn biochemistry and substantiates the highly welcoming attitudes of students towards this intervention.

### Limitations of the study

Our study is subjected to certain limitations. The evaluation of the METAPAD gaming puzzle has been done only with one batch of medical students thus participants size is low (*n* = 108). Secondly, the academic performance of students was not tested after the administration of this game-based learning using the METAPAD gaming puzzle as innovation. Also, a comparative study was not done using case and control groups to statistically prove academic performance is better in game based learning (gamification in medical education) when compared to traditional classroom teaching.

### Recommendations

Owing to the paucity in information on association between preferred learning styles, genre of games and its influence on academic performance of medical students, it is recommended that more studies should be conducted in this regard. Also, the practical implementation of such game based learning in academic calendar should be evaluated. Pre and post tests during game based learning will help in understanding the overall benefit from these innovations. Therefore future studies should also include ‘testing of knowledge’ as an essential component while evaluating a study tool.

## Conclusion

The METAPAD gaming puzzle has received highly welcoming responses from students clearly indicates gamification as an effective strategy for learning intricate metabolic pathways in biochemistry. This helps in overcoming the drawbacks of conventional classroom with student-centred learning methods that instil motivation and curiosity in students’ minds in order to understand and learn complex concepts with ease and in a more self-directed and self-evaluative manner. This is also an innovative approach for the faculties to design and develop constructive learning methodologies. Therefore, the METAPAD gaming puzzle enhances active learning of complex metabolic pathways in biochemistry by promoting learner interest and collaboration. METAPAD facilitates self-directed learning and improves the knowledge retention, critical and problem-solving abilities of students. Also, different types of learners have perceived METAPAD as an effective learning tool with an emphasis on the need to stay focused while solving the puzzle. Thus, learning style preferences do not greatly influence the perceptions towards METAPAD making it a universal tool for learning metabolic pathways. However, factors such as the extent of “concentration” or “focus” required to solve puzzles should be taken into consideration while preparing and implementing these kinds of games to adhere to the learning needs and styles of students. Future studies should investigate more on the implementation of the same on a wider scale and analyse the impact of game-based learning in medical education.

### Supplementary Information

**Additional file 1.**

## Data Availability

The data that supports this study are available upon request from the corresponding author.
