# Supplementary material for: “METAPAD” (METAbolic PAthways Decoded) – a gaming innovation to ease the complexity of metabolic pathways by promoting self-directed, active, participatory learning in small groups
Source: BMC Med Educ. 2023 Aug 25;23:608. doi: 10.1186/s12909-023-04587-5 (PMC10464076; doi:10.1186/s12909-023-04587-5)
Supplement: Supplementary file 1 — Additional file 1. [file 12909_2023_4587_MOESM1_ESM.pdf]

PANIMALAR
MEDICAL COLLEGE HOSPITAL
& RESEARCH INSTITUTE
MAKING A HEALTHY TOMORROW

LEARNER'S PERCEPTIONS ON "METAPAD (METAbolic PAthways Decoded)" GAMING PUZZLE EXCERCISES AS AN INNOVATIVE TOOL IN LEARNING BIOCHEMISTRY IN FIRST PROFESSIONAL MEDICAL CURRICULUM.

1.

This is to be filled by individual student (anonymous) following completion of the each "METAPAD (METAbolic PAthways Decoded)" gaming puzzle exercises as an innovative tool in learning biochemistry and handed over to the facilitator.

* 1. Please Enter your Date of Birth (DD/MM/YYYY)

Date / Time

Date

DD/MM/YYYY

* 2. Enter your Age

* 3. Gender

- Male
- Female
- Trans Male
- Trans Female
- Gender Variant / Non-conforming
- Not listed

* 4. Name of the College

* 5. Mention your Learning Prefernce as per the "VARK" Learning Style Inventory

- Visual
- Auditory
- Read/Write
- Kinaesthetic
- Multimodal

* 6. Course

◯ MBBS

PANIMALAR
MEDICAL COLLEGE HOSPITAL
& RESEARCH INSTITUTE
MAKING A HEALTHY TOMORROW

LEARNER'S PERCEPTIONS ON "METAPAD (METAbolic PAthways Decoded)" GAMING PUZZLE EXCERCISES AS AN INNOVATIVE TOOL IN LEARNING BIOCHEMISTRY IN FIRST PROFESSIONAL MEDICAL CURRICULUM.

2.

Please respond to these questions / statements regarding the "METAPAD (METAbolic PAthways Decoded)" gaming puzzle exercises as an innovative tool in learning biochemistry in Phase-I of undergraduate medical curriculum. For each one of the following objectives, could you give your opinion from your learning experience using the METAPAD gaming puzzle approach ?

* 1. I have enjoyed solving the METAPAD gaming Puzzles.

- ◯ Strongly agree
- ◯ Agree
- ◯ Neither agree nor disagree
- ◯ Disagree
- ◯ Strongly disagree

* 2. The clinical cases given in METAPAD gaming Puzzles were interesting.

- ◯ Strongly agree
- ◯ Agree
- ◯ Neither agree nor disagree
- ◯ Disagree
- ◯ Strongly disagree

* 3. METAPAD gaming Puzzle tool stimulates my curiosity in learning the complex metabolic pathways in biochemistry.

- ◯ Strongly agree
- ◯ Agree
- ◯ Neither agree nor disagree
- ◯ Disagree
- ◯ Strongly disagree

* 4. The METAPAD gaming Puzzle tool polishes my creativity.

- Strongly agree
- Agree
- Neither agree nor disagree
- Disagree
- Strongly disagree

* 5. The METAPAD gaming Puzzle tool enhances my problem-solving ability.

- Strongly agree
- Agree
- Neither agree nor disagree
- Disagree
- Strongly disagree

* 6. The METAPAD gaming Puzzles helped in improving your critical thinking ability?

- Strongly agree
- Agree
- Neither agree nor disagree
- Disagree
- Strongly disagree

* 7. We need to be focused to solve the METAPAD gaming Puzzle.

- Strongly agree
- Agree
- Neither agree nor disagree
- Disagree
- Strongly disagree

* 8. I am able to recall the topics while solving the METAPAD gaming Puzzle.

- Strongly agree
- Agree
- Neither agree nor disagree
- Disagree
- Strongly disagree

* 9. The METAPAD gaming Puzzles can be used to evaluate a student too.

- Strongly agree
- Agree
- Neither agree nor disagree
- Disagree
- Strongly disagree

* 10. METAPAD gaming Puzzle can increase interaction among the students.

- Strongly agree
- Agree
- Neither agree nor disagree
- Disagree
- Strongly disagree

* 11. The METAPAD gaming Puzzle enhanced my learning.

- Strongly agree
- Agree
- Neither agree nor disagree
- Disagree
- Strongly disagree

* 12. I have enjoyed classmate interaction and reviewing the content of metabolic pathways while solving the METAPAD gaming Puzzle.

- Strongly agree
- Agree
- Neither agree nor disagree
- Disagree
- Strongly disagree

* 13. METAPAD gaming Puzzle oriented us to the topics that we should focus on.

- Strongly agree
- Agree
- Neither agree nor disagree
- Disagree
- Strongly disagree

* 14. Length of time provided for solving the METAPAD gaming Puzzle was sufficient.

- Strongly agree
- Agree
- Neither agree nor disagree
- Disagree
- Strongly disagree

* 15. The material on the METAPAD gaming Puzzle was pertinent.

- Strongly agree
- Agree
- Neither agree nor disagree
- Disagree
- Strongly disagree

* 16. Remembering the enzyme and metabolite names was easier because of the METAPAD gaming Puzzle.

- Strongly agree
- Agree
- Neither agree nor disagree
- Disagree
- Strongly disagree

* 17. Solving METAPAD gaming Puzzle is a good review of the material covered in the lecture.

- Strongly agree
- Agree
- Neither agree nor disagree
- Disagree
- Strongly disagree

* 18. Extra credit should be associated with activities such as a METAPAD gaming Puzzle.

- Strongly agree
- Agree
- Neither agree nor disagree
- Disagree
- Strongly disagree

* 19. METAPAD gaming Puzzle is a good tool to study and revise the complex content of metabolic pathways.

- Strongly agree
- Agree
- Neither agree nor disagree
- Disagree
- Strongly disagree

* 20. METAPAD gaming Puzzle promoted meaningful learning instead of traditional classroom learning

- Strongly agree
- Agree
- Neither agree nor disagree
- Disagree
- Strongly disagree

* 21. METAPAD gaming Puzzle promotes active independent learning, student reasoning and communication skills

- Strongly agree
- Agree
- Neither agree nor disagree
- Disagree
- Strongly disagree

22. Do you wish to make any additional comments?
